# The Microstructure, Thermal, and Mechanical Properties of Sn-3.0Ag-0.5Cu-*x*Sb High-Temperature Lead-Free Solder

**DOI:** 10.3390/ma13194443

**Published:** 2020-10-07

**Authors:** Chaojun Li, Yanfu Yan, Tingting Gao, Guodong Xu

**Affiliations:** School of Materials Science and Engineering, Henan University of Science and Technology, Luo Yang 471000, China; llcj18437952530@163.com (C.L.); 18625557825@163.com (T.G.); 15237194370@163.com (G.X.)

**Keywords:** high temperature lead-free solder, mechanical behavior, microstructure, intermetallic compounds

## Abstract

To obtain Sn-3.0Ag-0.5Cu-*x*Sb (*x* = 0, 25, 28, and 31) high-temperature lead-free solder antimony was added to Sn-3.0Ag-0.5Cu solder. The microstructure, thermal properties, and mechanical behavior of the solder alloy prepared were studied by using JSM-5610LV scanning electron microscope, Germany STA409PC differential scanning calorimeter, AG-I250KN universal tensile testing machine, and other methods. The SEM-EDS results showed that after adding Sb, SnSb phase was formed in the β-Sn matrix phase. The newly formed SnSb phase and the existing Sb in the solder alloy can inhibit the generation of IMC and refine the IMC layer. The addition of Sb significantly increased the melting temperature of the solder alloy. Among them, the thermal performance of Sn-3.0Ag-0.5Cu-25Sb is the best. The melting temperature of Sn-3.0Ag-0.5Cu-25Sb is 332.91 °C and the solid–liquid line range of Sn-3.0Ag-0.5Cu-25Sb solder alloy is 313.28–342.02 °C. Its pasty range is 28.74 °C, lower than 30 °C, which is beneficial for soldering. The test results of the mechanical behavior of Sn-3.0Ag-0.5Cu-*x*Sb solder alloy show that with the increase of Sb addition, the ultimate tensile strength of the solder alloy also increases. However, the change of the elongation of the solder alloy is the opposite. The ultimate tensile strength of the solder alloy increased from 29.45 MPa of Sn-3.0Ag-0.5Cu solder to 70.81 MPa of Sn-3.0Ag-0.5Cu-31Sb solder. The reason for the increase in the strength of the solder alloy is the reduction of the thickness of IMC and the solid solution hardening effect of Sb.

## 1. Introduction

In the past few years, the solder of Sn-Pb has been used in the electronics industry on a large scale because of its unique properties, such as lower cost, better wettability, and ideal mechanical properties [1,2]. However, lead is very harmful to the human body and the environment, and its use is prohibited [3,4,5]. Therefore, the electronics industry needs new solder alloys to replace the solder of Sn-Pb. Among the alloy systems reported by the research team, the more promising is tin-based solder alloys [6,7,8,9]. In recent years, the research on Sn-Ag-Cu (SAC) solder alloys with high silver content has increased exponentially, because they usually have good mechanical behavior and good welding ability, making them a promising material for microelectronic applications [10,11,12,13,14]. However, SAC solder alloy also has the defects of low melting point and short creep rupture life, which cannot replace high-temperature tin-lead solder [15]. Studies on high-temperature lead-free solder with melting point above 300 °C are imminent.

Accordingly, adding many alloy elements, like In, Ni, Sb, Ga, etc., to Sn-Ag-Cu solder allows obtaining a high-temperature lead-free solder that has good thermal and mechanical behavior [16,17,18,19]. According to some research studies, the addition of Sb and other elements could obtain good mechanical properties. The reasons for the improvement are the solid solution hardening effect of Sb, the hardening effect of the SnSb phase and the existence of Ag_3_Sn intermetallic compounds in the β-Sn matrix [7,20]. Research also showed that the addition of Sb could inhibit the formation of coarse β-Sn and refine the precipitation of Ag_3_Sn, thereby improving the mechanical and thermal behavior of Sn-Ag-Sb solder [8,20]. The microstructure changes of Sn-Ag solder alloys added with Sb were studied by Lee et al. [21]. The results showed that antimony changed the microstructure of Sn-3.5Ag, resulting in solid solution hardening. The addition of Sb can refine the needle-shaped Ag_3_Sn compound and improve the hardness, bonding strength, and thermal performance of the Sn-3.5Ag solder joint. Moreover, the addition of 1.5 wt.% Sb could narrow the temperature range of solidus and liquidus, which is an ideal result for actual welding process. Phairote and Thawatchai found that after adding Sb, the microstructure of the alloy is improved, the IMC distribution is more uniform, and new Ag_3_(Sn, Sb) and Cu_6_(Sn, Sb)_5_ IMC are formed around the β-Sn phase [7]. Chen and Li added Sb to the Sn-Ag-Cu solder to prepare a Sn-3.5Ag-0.7Cu-1Sb alloy. It was found that Ag_3_(Sn, Sb) intermetallic compound layer was formed, and the thickness and size of the intermetallic compound were reduced [22,23]. The study of Mahmudi and Mahin-Shirazi [1] showed that the tensile strength and plasticity of solder alloys could be improved by adding 1.5 wt.% Sb to Sn-3.5Ag alloys. The solid solution hardening effect of Sb increased the strength. This increase in plasticity is due to the refinement of the alloy structure. SnSb and Ag_3_Sn intermetallic compounds have important effects on the mechanical properties of Sn-Sb solders, such as tensile strength and shear resistance, because they act as obstacles to dislocation movement [24]. This study studied the thermal properties, microstructure, and mechanical behavior of Sn-3.0Ag-0.5Cu-*x*Sb high-temperature lead-free solder alloy.

## 2. Materials and Methods

In order to reduce the impurity content in the solder alloy, the raw materials used in the preparation of the solder alloy are 99.95% (purity) tin, 99.99% copper, 99.99% silver, and 99.95% antimony. The solder alloy is prepared by melting in an intermediate frequency induction heating furnace, and sodium phosphate is used as an anti-oxidation protective agent during melting. The composition of the solder alloy is listed in Table 1.

Before testing the microstructure of the alloy, the alloy was sampled, ground, polished, and corroded with a 5% HNO_3_, 2% HCl, and 93% CH_3_OH solution for 30 s. The microscopic morphology of the solder alloy was observed with JSM-560 LV scanning electron microscope (SEM). The phase composition and element diffusion were measured with an energy spectrometer (EDS) and a JSM-5610LV scanning electron microscope. The melting point of the solder alloy was measured by differential thermal analysis. A Shimadzu AG-I250KN precision universal tensile testing machine was used to test the ultimate tensile strength of the alloy. The tensile test was performed at a constant speed of 1.0 mm/min at room temperature. Three samples were tested for each ingredient, and the average value was used as the final tensile strength. The microhardness of the sample was measured with a Vickers hardness tester, measured ten times, and the average value was taken as the test result.

## 3. Results and Discussion

### 3.1. Microstructure of Solder Alloy

**Figure 1:**
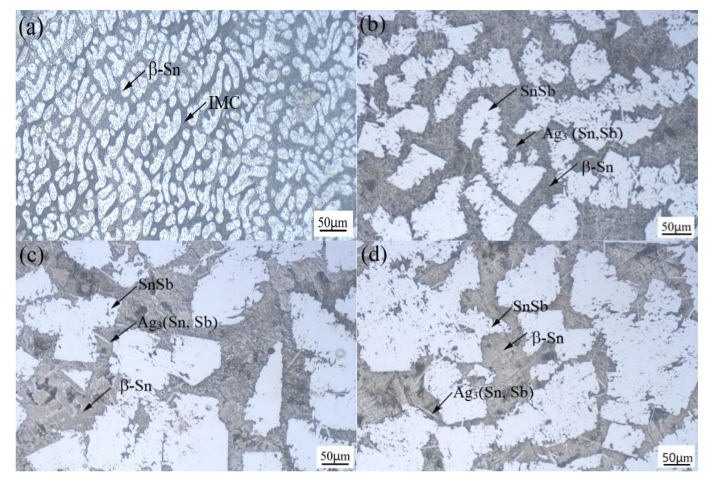
The microstructure of the Sn-3.0Ag-0.5Cu-xSb solder alloy: (**a**) *x* = 0, (**b**) *x* = 25, (**c**) *x* = 28, and (**d**) *x* = 31.

**Figure 2:**
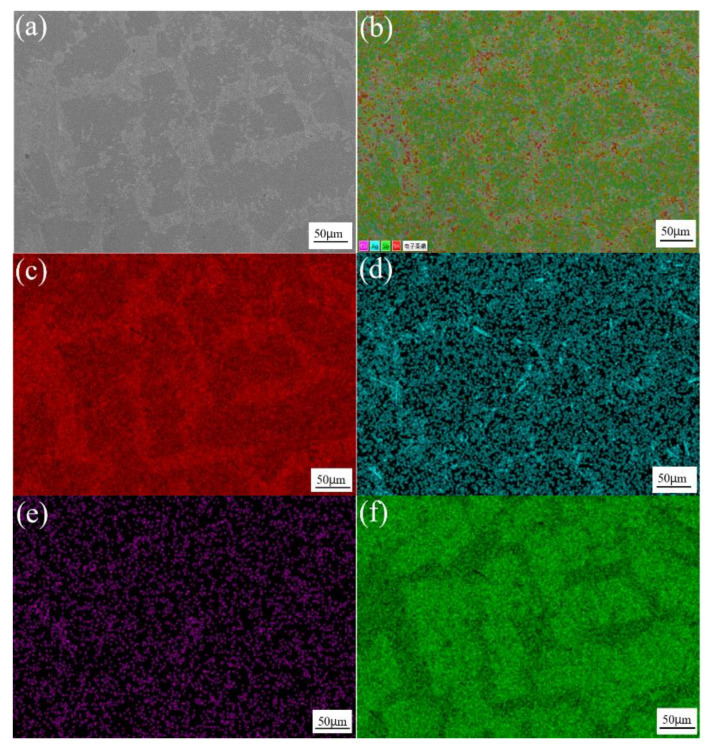
SEM image at 200x of Sn-3.0Ag-0.5Cu-25Sb (**a**); combined elemental map (**b**); and element distribution maps of Sn (**c**), Ag (**d**), Cu (**e**), and Sb (**f**).

Figure 1 shows the microstructure of the Sn-3.0Ag-0.5Cu-*x*Sb (*x* = 0, 25, 28 and 31) solder alloy. In Figure 1a, we can observe that the microstructure of the Sn-3.0 Ag-0.5Cu solder alloy is composed of two parts: a brighter area and a darker matrix area. The brighter bulk phase is the β-Sn phase. The darker matrix region contains the intermetallic compound phase (Ag_3_Sn phase, Cu_6_Sn_5_ phase) and β-Sn phase. Refer to the Sn-Sb binary alloy phase diagram [25], Sn_3_Sb_2_ phase is formed when the temperature drops to 324 °C. However, the Sn_3_Sb_2_ phase is an unstable phase [26,27]. When the temperature is lower than 242 °C, Sn_3_Sb_2_ will decompose into SnSb phase and β-Sn phase. Figure 1b–d shows the microstructure of the solder alloy after adding element Sb. From these SEM images, it can be clearly seen that there are white bulk phases distributed in the matrix. Combining the element distribution diagram of Sn-3.0Ag-0.5Cu-25Sb in Figure 2 with that in [1,7], it can be determined that the white bulk phase is the SnSb phase and the matrix is the β-Sn phase. With the increase of Sb content, the white bulk SnSb phase gradually becomes larger, IMCs become thinner, and the acicular intermetallic compound increases and pinches along the grain boundary. According to previous reports [7,23], the needle-like intermetallic compounds are Ag_3_Sn and Ag_3_(Sn, Sb). These were confirmed in the subsequent EDS analysis. Ag_3_Sn and Ag_3_(Sn, Sb) intermetallic compounds are brittle phases with low plasticity, which will affect the mechanical properties and thermal fatigue life of the solder joints [20,22]. It can also be found from the SEM image that an appropriate amount of Sb can inhibit the coarsening of Ag_3_Sn and Ag_3_(Sn, Sb) intermetallic compounds, thereby improving the mechanical behavior of the solder joints.

**Figure 3:**
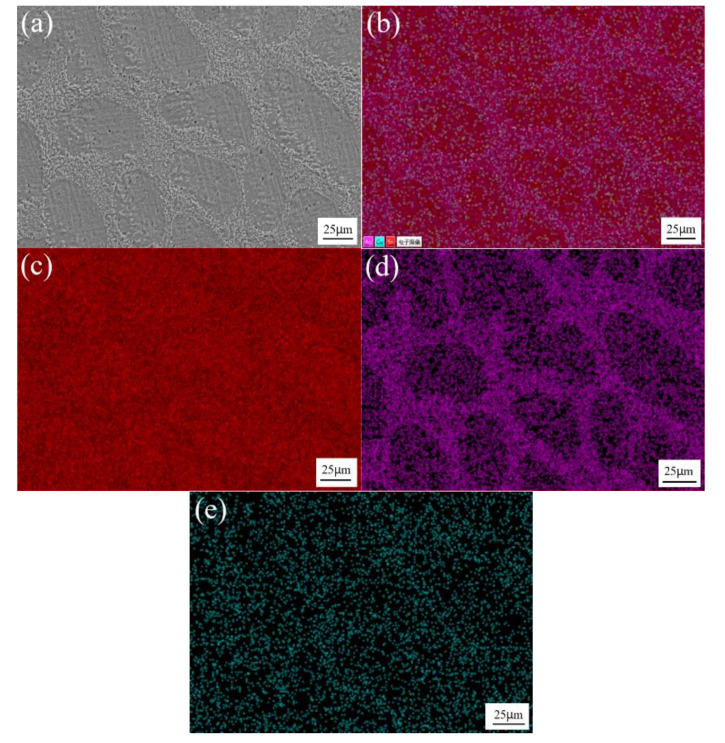
SEM image at 500x of Sn-3.0Ag-0.5Cu (**a**); combined elemental map (**b**); and element distribution maps of Sn (**c**), Ag (**d**), and Cu (**e**).

**Figure 4:**
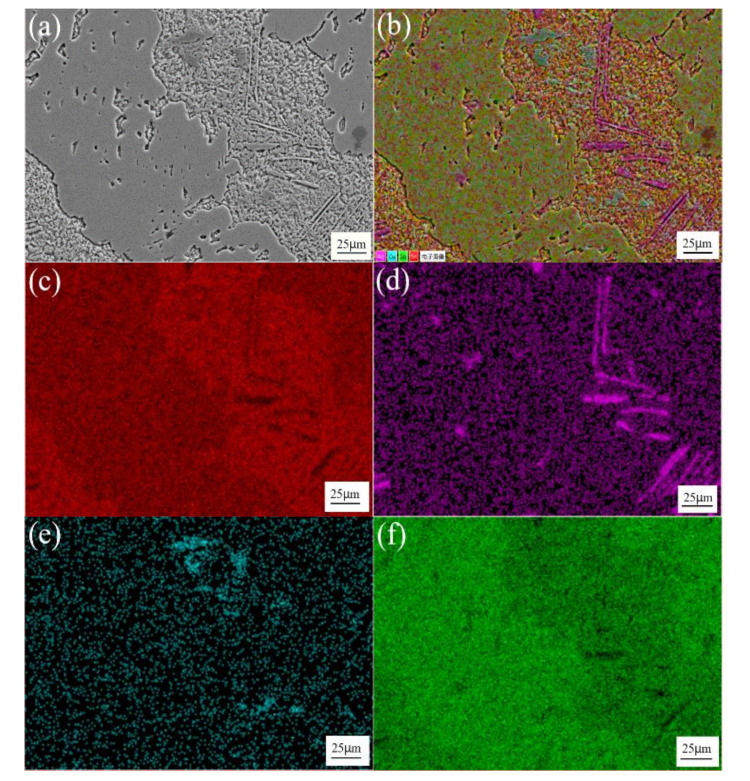
SEM image at 500x of Sn-3.0Ag-0.5Cu-25Sb (**a**); combined elemental map (**b**); and element distribution maps of Sn (**c**), Ag (**d**), Cu (**e**), and Sb (**f**).

Figure 3 shows the SEM image and element distribution maps of Sn-3.0Ag-0.5Cu solder alloys. As can be seen in the figure, the red area is the β-Sn matrix phase. The purple and dark green areas are Ag and Cu element distribution areas, and Ag_3_Sn and Cu_6_Sn_5_ IMCs are generated in these areas, which are distributed along the β-Sn matrix. Figure 4 shows the SEM image and element distribution results of Sn-3.0Ag-0.5Cu-25Sb solder alloy. The red area shows the Sn element distribution, and the purple area shows Ag. The dark green area and the green area are Cu and Sb elements. The Ag-rich phase and Cu-rich phase are distributed in the β-Sn matrix phase to form IMCs. A large amount of Sn and Sb are distributed in the dark gray massive area, forming the SnSb phase.

**Figure 5:**
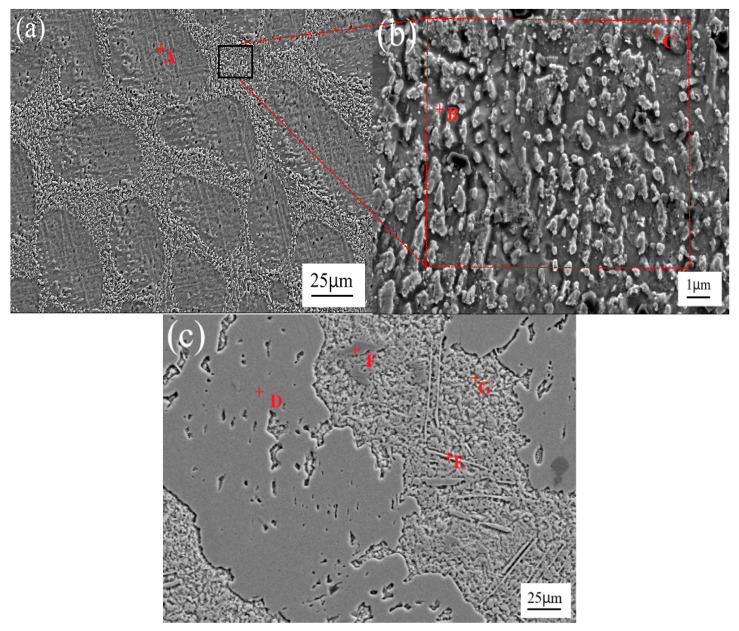
EDS analysis of (**a**,**b**) Sn-3.0Ag-0.5Cu solder alloy and (**c**) Sn-3.0Ag-0.5Cu-25Sb solder alloy.

Figure 5 shows the EDS analysis positions of Sn-3.0Ag-0.5Cu and Sn-3.0Ag-0.5Cu-25Sb solder alloys. Among them, Figure 5b is an enlarged view of the partial position of Figure 5a. The EDS analysis results are listed in Table 2. It confirms that Sn-3.0Ag-0.5Cu is composed of β-Sn phase, needle-shaped Ag_3_Sn, and scallop-shaped Cu_6_Sn_5_ IMCs phase. In Figure 5c, we can observe that Sn-3.0Ag-0.5Cu-25Sb contains SnSb phase, β-Sn phase, Cu_6_(Sn,Sb)_5_ phase, and Ag_3_(Sn,Sb) phase. The formation of SnSb phase and the presence of Sb in the alloy can inhibit the growth of intermetallic compounds, thereby improving the mechanical behavior of the solder alloy [20].

### 3.2. DSC Analysis of the Solder Alloy

**Figure 6:**
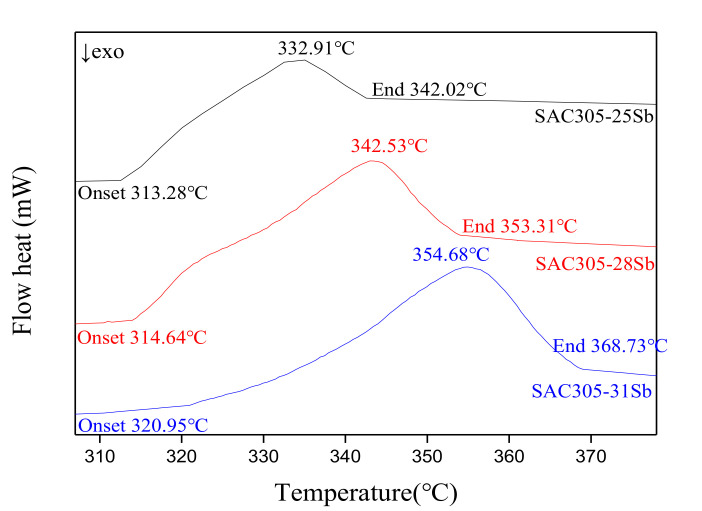
The direct scanning calorimetry (DSC) curve of the Sn-3.0Ag-0.5Cu-*x*Sb (*x* = 25, 28, 31) solder alloy.

In the process of semiconductor device packaging, the soldering of insulating substrates and leads, the soldering of chips and leads, and the soldering of shell packaging and other chip packaging, the solder used for connection cannot be melted in the next process. Therefore, it is necessary to use higher melting point and lower melting point solder in the semiconductor packaging process. The melting point of Sn-3.0Ag-0.5Cu-*x*Sb (*x* = 25, 28, 31) is required to be above 300 °C. The melting temperature of the experimental alloy was measured with a Germany STA409PC differential scanning calorimeter, and the heating rate was 10 °C/min. Figure 6 shows the differential scanning calorimetry (DSC) curve of the Sn-3.0Ag-0.5Cu-*x*Sb (*x* = 25,28 and 31) solder alloy. Table 3 lists the results of the DSC heating curve. Obviously, as the amount of Sb added increased, the melting temperature and pasty range of the solder alloy also increased. This result is consistent with previous reports about the addition of Sb to Sn-Ag solder alloy [28,29]. The melting temperatures of Sn-3.0Ag-0.5Cu, Sn-3.0Ag-0.5Cu-25Sb, Sn-3.0Ag-0.5Cu-28Sb, Sn-3.0Ag-0.5Cu-31Sb solder alloys were 218.90, 332.91, 342.53, and 354.68 °C, respectively. The melting point of Sb-containing solder alloy was much higher than that of Sn-3.0Ag-0.5Cu, which is due to the formation of bulk SnSb phase. The pasty ranges of Sn-3.0Ag-0.5Cu, Sn-3.0Ag-0.5Cu-25Sb, Sn-3.0Ag-0.5Cu-28Sb, Sn-3.0Ag-0.5Cu-31Sb solder alloy were 5.19, 28.74, 38.64, and 47.78 °C, respectively. The pasty range of the solder was significantly increased with the addition of Sb. Generally, solder alloys with a small pasty range have good soldering properties. However, according to the lead-free solder project of the National Center for Manufacturing Sciences (NCMS), it is recommended that the pasty range of the new lead-free solder should not exceed 30 °C [30]. Therefore, the Sn-3.0Ag-0.5Cu-25Sb solder alloy can be used as a high-temperature lead-free solder for soldering because its melting temperature is higher than 300 °C and the pasty range is lower than 30 °C.

### 3.3. Mechanical Properties of Solder Alloy

**Figure 7:**
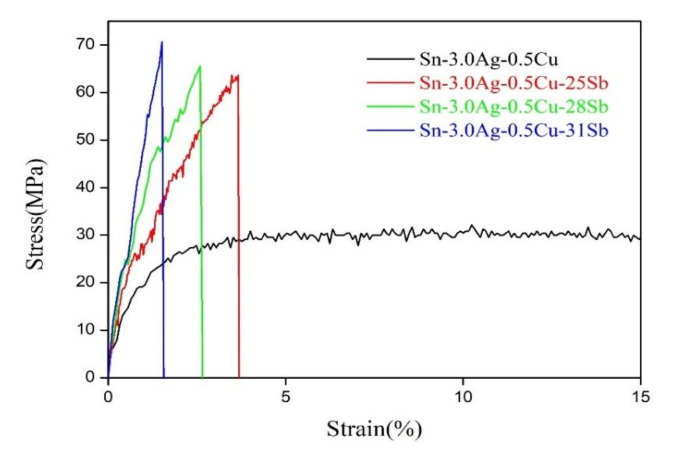
Stress–strain curves of Sn-3.0Ag-0.5Cu-*x*Sb solder alloy.

**Figure 8:**
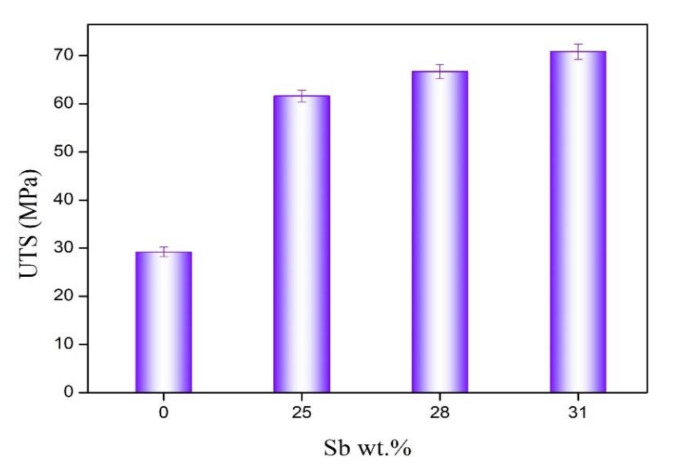
Ultimate tensile strength of Sn-3.0Ag-0.5Cu-*x*Sb solder alloy.

Figure 7 shows the stress–strain curve of the solder alloy. Obviously, the increase in strength and decrease in plasticity of the solder alloy can be seen. Figure 8 is the result of the ultimate tensile strength of the solder alloy. The results in Figure 8 show that as the Sb content increases, the ultimate tensile strength of the alloy also increases. The ultimate tensile strength of the alloy increased from 29.45 MPa of Sn-3.0Ag-0.5Cu solder to 70.81 MPa of Sn-3.0Ag-0.5Cu-31Sb solder. It is noteworthy that after adding 25% Sb to Sn-3.0Ag-0.5Cu, the ultimate tensile strength reached 61.62 MPa, which is twice higher than the ultimate tensile strength of Sn-3.0Ag-0.5Cu. There are two reasons for the increase in ultimate tensile strength of Sn-3.0Ag-0.5Cu-*x*Sb (*x* = 0, 25, 28 and 31). One is the reduction of the thickness of the intermetallic compound in the solder alloy. Studies have shown that SnSb and Ag_3_Sn IMC act as a hindrance to the movement of dislocations, which has an important impact on creep resistance and the tensile strength of the solder alloy [3]. Chen found that the addition of Sb in the Sn-Ag-Cu alloy can significantly inhibit the formation of intermetallic compounds. It can also reduce the element diffusion caused by the grain boundary pinning effect during the aging process [20]. In addition, G.Y. Li also confirmed that the greater the thickness of the IMC, the lower the tensile strength [23]. Another reason is the solid solution hardening effect of Sb. Some studies have found that when Sb is added to SAC alloy, Sb can have a solid solution hardening effect in the β-Sn matrix phase [31,32]. After adding Sb, more Ag_3_(Sn, Sb) and Cu_6_(Sn, Sb)_5_ particles precipitated on the Cu_6_Sn_5_ layer, and the grain hardening effect occurred [7].

**Figure 9:**
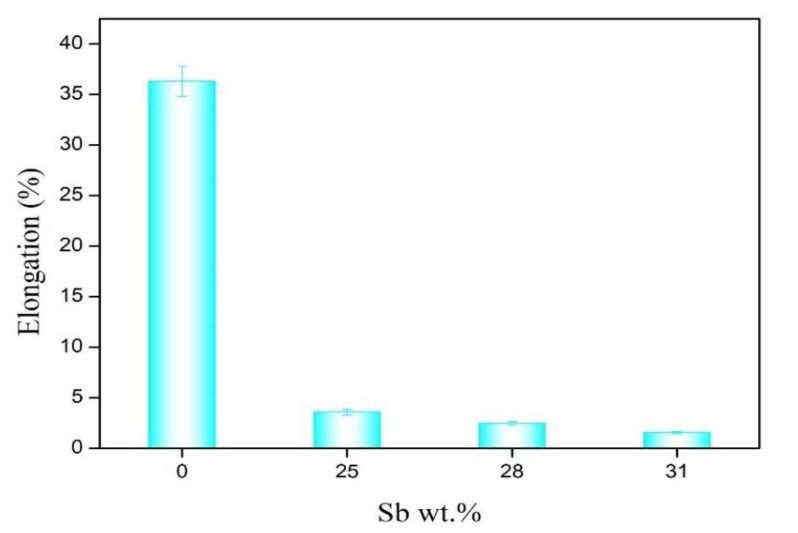
The elongation of Sn-3.0Ag-0.5Cu-*x*Sb solder alloy.

Figure 9 shows the elongation of the alloy. Obviously, as the increase of Sb addition, the elongation of the solder alloy is greatly reduced. After adding 25% Sb, the elongation of the solder alloy decreased from 37.20% of Sn-3.0Ag-0.5Cu solder to 3.59% of Sn-3.0Ag-0.5Cu-25Sb solder. Therefore, with increasing Sb addition, the ultimate tensile strength of the solder alloy increases and the elongation decreases, which is consistent with the results of some studies [7,33].

**Figure 10:**
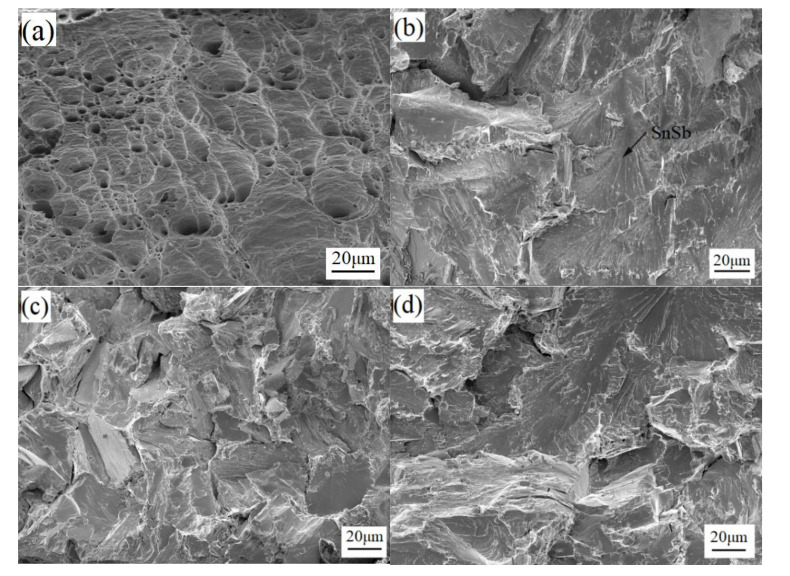
Microscopic fracture surface of (**a**) Sn-3.0Ag-0.5Cu, (**b**) Sn-3.0Ag-0.5Cu-25Sb, (**c**) Sn-3.0Ag-0.5Cu-28Sb, and (**d**) Sn-3.0Ag-0.5Cu-31Sb.

Figure 10 is the microscopic fracture surface of the solder alloy. In Figure 10a, it can be observed that the microscopic fracture surface of Sn-3.0Ag-0.5Cu has obvious dimple morphology, and dimple is an important feature of microvoid coalescence fracture. This shows that the solder alloy has undergone strong plastic deformation before fracture, and the fracture mode is plastic fracture. In Figure 10b–d, a fan shape can be observed, indicating that the solder alloy is cleavage fracture, which is brittle fracture. Under stress, the cracks start to grow from the brittle SnSb phase [34]. When the crack propagates to the grain boundary, the larger orientation difference between adjacent grains prevents the crack propagation. At this time, the stress concentration at the crack tip can be released in adjacent grains, causing new cleavage crack nucleation. Therefore, the cleavage crack cannot expand through the grain boundary to form a river pattern, but forms a fan shape and continues to expand. The microscopic fracture surface of the solder alloy shows that after adding Sb, the fracture mode of the solder alloy changes from the original ductile fracture to the brittle fracture. This is because the addition of Sb causes the solder alloy to generate many brittle SnSb phases and needle-like Ag_3_(Sn, Sb) phases.

**Figure 11:**
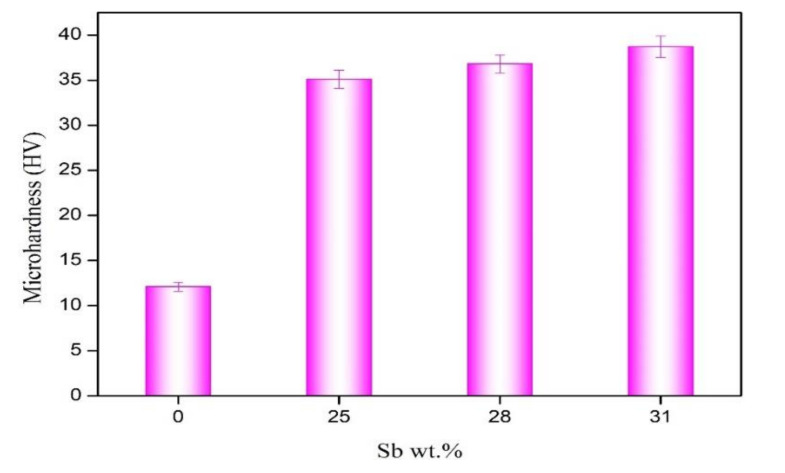
Microhardness of Sn-3.0Ag-0.5Cu-*x*Sb solder alloy.

Figure 11 shows the microhardness of the solder alloy. After adding Sb, the microhardness of the solder alloy was greatly improved. The microhardness of Sn-3.0Ag-0.5Cu is 12.1 HV. The microhardness of Sn-3.0Ag-0.5Cu-31Sb is 38.7 HV, which is three times more than Sn-3.0Ag-0.5Cu. This is because the solid solution strengthening effect of Sb increase the microhardness of the solder alloy.

## 4. Conclusions

(1) The microstructure study showed that after adding Sb, Sn-Sb phase, Cu_6_(Sn,Sb)_5_ phase, and Ag_3_(Sn,Sb) phase were formed in the solder alloy. The Ag_3_(Sn,Sb) phase and Cu_6_(Sn,Sb)_5_ phase are distributed in the β-Sn matrix phase. The addition of Sb suppresses and refines the formation of intermetallic compounds and improves the microstructure of the solder alloy.
(2) The addition of Sb significantly increases the melting point and paste range of the Sn-3.0Ag-0.5Cu-*x*Sb solder alloy. The melting point of the solder alloy increases with the increase of Sb. Among them, the thermal performance of Sn-3.0Ag-0.5Cu-25Sb is the best. The melting temperature of Sn-3.0Ag-0.5Cu-25Sb is 332.91 °C, and the solid–liquid temperature is 313.28–342.02 °C. The pasty range is 28.74 °C, which is lower than the 30 °C recommended by NCMS.
(3) With the increase of Sb addition, the ultimate tensile strength of Sn-3.0Ag-0.5Cu-*x*Sb (*x* = 0, 25, 28, and 31) solder alloy increases, while the elongation decreases. The ultimate tensile strength of the solder alloy increased from 29.45 MPa of Sn-3.0Ag-0.5Cu solder to 70.81 MPa of Sn-3.0Ag-0.5Cu-31Sb solder. The increase in the ultimate tensile strength of the solder alloy is due to the reduction in the thickness of IMC by Sb and solid solution hardening effect of Sb. The solder alloy changes from plastic fracture to brittle fracture.
(4) The microhardness of the solder alloy is greatly improved. The microhardness of Sn-3.0Ag-0.5Cu is 12.1 HV at 100 g and the microhardness of Sn-3.0Ag-0.5Cu-25Sb is 35.1 HV at 100 g.

In summary, considering the thermal and mechanical behavior, the optimal addition of Sb in the solder alloy is 25 wt.% in this study.

## Figures and Tables

**Table 1:** Composition of solder alloy (wt.%).

Materials	Sn	Ag	Cu	Sb
Sn-3.0Ag-0.5Cu	balance	3.11	0.48	—
Sn-3.0Ag-0.5Cu-25Sb	balance	3.24	0.47	25.09
Sn-3.0Ag-0.5Cu-28Sb	balance	3.18	0.51	28.17
Sn-3.0Ag-0.5Cu-31Sb	balance	3.35	0.47	31.21

**Table 2:** Energy spectrometer (EDS) analysis results of marked points (wt.%).

Position	Element				Possible Composition
	Sn	Ag	Cu	Sb	
A	99.00	0.41	0.59	—	β-Sn
B	24.65	75.35	—	—	Ag_3_Sn
C	65.73	—	34.27	—	Cu_6_Sn_5_
D	53.39	—	—	46.61	SnSb
E	23.36	70.21	—	6.43	Ag_3_ (Sn,Sb)
F	60.51	—	33.69	5.80	Cu_6_ (Sn,Sb)_5_
G	88.93	—	—	11.07	β-Sn

**Table 3:** The solidus temperature (Tonset), liquidus temperature (Tend), and melting temperature of solder alloy from DSC heating curve.

Solder Alloys	Tonset (°C)	Tend (°C)	Pasty Range (Tend–Tonset) (°C)	Melting Temperature(°C)
Sn-3.0Ag-0.5Cu	216.32	221.51	5.19	218.90
Sn-3.0Ag-0.5Cu-25Sb	313.28	342.02	28.74	332.91
Sn-3.0Ag-0.5Cu-28Sb	314.64	353.31	38.67	342.53
Sn-3.0Ag-0.5Cu-31Sb	320.95	368.73	47.78	354.68
